# Prevalence of renal dysfunction among HIV infected patients receiving Tenofovir at Mulago: a cross-sectional study

**DOI:** 10.1186/s12882-020-01873-y

**Published:** 2020-06-22

**Authors:** Louis Nyende, Robert Kalyesubula, Emmanuel Sekasanvu, Pauline Byakika-Kibwika

**Affiliations:** 1 KCCA- directorate of public health, P.O. Box 7010, Kampala, Uganda; 2 School of Medicine, Department of Internal Medicine, Makerere University College of Health Sciences, Kampala, Uganda; 3 Panorama medical centre, Kampala, Uganda

**Keywords:** Renal dysfunction, Tenofovir disproxil fumarate, Estimated glomerular filtration rate

## Abstract

**Background:**

There is an increasing burden of non-communicable disease globally. Tenofovir disoproxil fumarate (TDF) is the most commonly prescribed antiretroviral drug globally. Studies show that patients receiving TDF are more prone to renal dysfunction at some point in time during treatment. Evaluation of kidney function is not routinely done in most HIV public clinics. Identification of renal dysfunction is key in resource constrained settings because managing patients with end stage renal disease is costly.

**Method:**

This was a cross-sectional study conducted at an outpatient clinic in 2018 involving patients on TDF for at least 6 months who were 18 years or older. Patients with documented kidney disease and pregnancy were excluded. Estimated glomerular filtration rate (eGFR) was calculated using the CKD-Epi formula. Renal dysfunction was defined as any of the following; either eGFR< 60 mL/min/1.73m^2^,or proteinuria of ≥2^+^ on urine dipstick, glycosuria with normal blood glucose. Electrolyte abnormalities were also documented.

**Results:**

We enrolled 278 participants. One hundred sixty nine (60.8%) were females, majority 234(84.2%) were < 50 years old, 205 (73.74%) were in WHO stage 1, most participants 271(97.5%) in addition to TDF were receiving lamivudine/efavirenz. The median age was 37(IQR 29–45) years; median duration on ART was 36 (IQR 24–60) months. The prevalence of renal dysfunction was 2.52% (7/278). Most noted electrolyte abnormality was hypocalcaemia (15.44%).

**Conclusions:**

The prevalence of renal dysfunction was low though some participants had hypocalcaemia. Screening for kidney disease should be done in symptomatic HIV infected patients on TDF.

## Background

People living with HIV (PLHIV) are living longer and as a result face a challenge of increasing morbidity from non-communicable diseases-NCDS [1, 2]. Kidney disease manifesting as renal dysfunction is one of the emerging NCDs in the world, with a varying prevalence of 13.9 to 48.5% [3, 4] . There is increasing concern that HIV being epidemic in this region, is contributing to the increasing prevalence of renal dysfunction in Sub-Saharan Africa [3] The etiology of renal dysfunction includes; HIV-associated nephropathy (HIVAN), HIV immune complex kidney disease (HIVICK), drugs used for treatment of opportunistic infections, antiretroviral therapy (ART),use of non-steroidal anti-inflammatory drugs (NSAIDS),herbal medicines, other co morbid conditions namely diabetes mellitus, hypertension and Hepatitis B & C. The consequences of renal dysfunction range from acute kidney injury (AKI), chronic kidney disease, end stage renal disease (ESRD) or death [5]. In countries with limited resources curative services for NCDs are an obvious strain to government rendering prevention as the best strategy [4, 6, 7]. Prevention involves identifying those conditions early, continuous health education/screening of the affected people and appropriate referral for proper management to delay complications.

The 90–90-90 UNAIDS strategy by 2020 [8] is not far away, but with it more people will be on ART coupled with the “test and treat approach”, whereby an individual testing positive for HIV is initiated on ART regardless of the CD4 or WHO clinical stage [9]. People living with HIV will be on ART for a long time yet with advancing age renal dysfunction deteriorates [10]. When ART programs started in Uganda, there was a lot of emphasis on initial evaluation of patients before starting treatment, but as the number of patients has increased this has not always been the case [11]. With regard to ART, patients on Tenofovir disproxil fumarate (TDF)based regimen from a number of studies are more prone to decline in kidney function. Other factors associated with renal dysfunction include, being older, being female, African-American ethnicity, low weight, low CD4 count, high viral load and having co-morbid conditions [12]. The cost of managing a patient with ESRD is high yet early identification with simply estimating glomerular filtration rate and a urine dipstick can help. The problem is even made worse by presentation of renal dysfunction being asymptomatic, late patient presentation, having few nephrologists and few/costly renal replacement therapies [13]. There are few studies quantifying the magnitude of renal dysfunction among HIV positive patients in sub-Saharan Africa and no renal registries/inadequate data in the region [14, 15]. This study aimed to quantify the magnitude of renal dysfunction among HIV infected patients receiving TDF based ART.

## Methods

### Study site

The study was carried out at the Mulago Communicable Disease Clinic under the directorate of medicine situated in Kampala, Uganda. Mulago-Kiruddu hospital is home to the directorate of medicine since May 2016; running different medical outpatient and inpatient clinics. The communicable disease clinic is supported by Makerere University Joint AIDS Program (MJAP), runs on Fridays as an outpatient clinic, and mainly attends to HIV infected patients. All services are provided free of charge to the public.

### Study design

We conducted a cross sectional study with the sample size calculated based on the Kish and Leslie formula for prevalence studies. The sample size was calculated as 396 (based on prevalence of 18.6,Wantakisha et al) [16] at a precision α of 0.05.We included adults aged 18 years and above on TDF based ART for at least 6 or more months. Participants were enrolled consecutively for convenience of which 278 met the inclusion criteria. Reasons for exclusion included; being on TDF for less than 6 months, pregnancy and refusal to join the study (Reasons given included; limited time, need to consult spouse, no interest in participating in the study and some were already in other studies).

### Study procedures

Patients, who fulfilled the study criteria were enrolled, interviewed for demographic and social data. Medical history was collected and physical examination performed. Blood pressure measurement was done in standard way (after patient had rested for 5 min, in sitting position and two measurements were taken). Patients with a value greater than 140/90 mmHg were considered hypertensive. Blood pressure > 140 mmHg was considered systolic hypertension and a value of > 90 mmHg considered diastolic hypertension. Nutritional assessment was done using body mass index (BMI), obtained using the formula body weight in kilograms divided by height squared in meters. Venous blood was collected for renal function test and electrolytes (serum creatinine, urea, potassium, calcium and phosphate). Reference laboratory ranges for electrolytes;

- Potassium 3.5–5.0 mmol/l
- Calcium 2.2–2.7 mmol/l
- Phosphate 0.97–1.45 mmol/l

Results for the electrolytes were reported as hypo or hyper depending on these reference ranges.

Following aseptic technique, 10 mL of venous blood was drawn from each participant into a syringe. Seven mills (7mls) (of the 10mls) of blood for renal function tests & electrolytes (potassium, calcium phosphate) was placed in a red top vacutainer and centrifuged. Four mls of serum were then put in crial vials. The remaining 3 mls (of the 10 mL) were placed in a grey vacutainer bottle (to determine blood sugar). All these were adequately packaged in Ziploc bags and carrier for processing in old Mulago clinical laboratory. Renal function tests, electrolytes and blood sugar were determined using an automated Abott Machine, Architect plus-Ci Chemistry and immunochemistry analyzer.

Each participant was provided with a sterile urine container and educated briefly about provision of 20 mL of a mid-stream urine which was analyzed using a dipstick on the same day to determine presence of leucocytes, nitrites, hematuria or glucose. Estimated glomerular filtration rate (eGFR) was calculated using the Chronic kidney Disease Epidemiology Collaboration (CKD-EPI) formula which is more accurate than the Cockcroft gault formula [17]. For this study renal dysfunction was noted as either eGFR less than 60 ml/min/1.73m^2^or proteinuria of ≥2^+^ with glycosuria but normal blood sugar. As per Kidney foundation’s kidney Disease outcome quality Initiative (K/DOQI),eGFR< 60 for 3 months implies chronic kidney disease [6]. To capture patients of TDF nephrotoxicity we assessed proteinuria,glucosuria and serum glucose [18].

### Statistical analysis

Data was analyzed using STATA 14.0 software package. Continuous variables were summarized as means and standard deviation for normally distributed data, medians and interquartile ranges for not normally distributed data. Categorical variables were summarized as frequencies, percentages and also in tables and figures. Tests for the significance of association were made using the Pearson chi-square (*X*^2^) test for categorical variables and independent sample t test for continuous variables. The outcome variable was renal dysfunction.

## Results

From November 2017 through March 2018 (for 4 months), eight hundred eight (808) patients were consecutively screened in Mulago Communicable Disease Clinic for study enrolment. Three hundred five (305) patients met the eligibility criteria and were enrolled. Of these, 278 were entered into the final analysis and 27 patients were excluded due to incomplete data. One hundred sixty nine (60.8%) were female, majority 234 (84.2%) were < 50 years old. Median age was 37 (IQR 29–45) years. One hundred nineteen (42.81%) were married, 141(50.72%) had a primary education, 250(89.93%) had formal employment, 252(83.45%) resided in Kampala and 81 (29.14%) consumed alcohol. (Table 1).

**Table 1:** Socio-demographic Characteristics of the study participants receiving TDF based ART ≥6 months at Mulago Communicable disease clinic

Socio-demographics	Frequency(n)	Percentage (%)
Gender		
Male	**109**	**39.21**
Female	169	60.79
Age (Years) median,(IQR)	**37 (29–45)**	
18–34	**128**	46.04
35–49	**106**	38.13
≥ 50	**234**	84.17
Marital status		
Single	51	18.35
Married	119	42.81
Separated	85	30.58
Widowed	23	8.27
Education level		
Primary	141	50.72
Secondary	115	41.37
Tertiary	22	7.91
Occupation		
Unemployed	28	10.07
Employed	250	89.93
Address		
Outside Kampala	46	16.55
Within Kampala	232	83.45
Alcohol		
No	197	70.86
Yes	81	29.14

### Clinical and laboratory characteristics (Table 2)

The median duration on ART was 36 IQR (24–60) months, 271 (97.48%) were on TDF/3TC/EFV, 152(72.38%) had CD4 ≥ 200 cells/ml, 109 (86.51%) had viral load of < 1000 copies/ml, 205(73.74%) were in WHO clinical one,155(55.76%) had a normal BMI, 38(13.67%) had systolic hypertension, 42(15.11%) had diastolic hypertension and 270(97.12%) had no concurrent opportunistic infections. Three (1.08%) had random blood sugar ≥11.1 mmol/l, 10 (3.6%) had proteinuria of ≥2^+^, 4(1.44%) tested positive for nitrites, 7(2.52%) had hematuria and 2(0.72%) had glycosuria.

**Table 2:** Clinical characteristics of the study participants receiving TDF based ART ≥6 months at Mulago Communicable Disease Clinic

Characteristics	Frequency(n)	Percentage (%)
BMI		
18.5–24.9-Normal	155	55.76
25–29.9- Overweight	78	28.06
≥ 30 - Obese	24	8.63
Systolic hypertension		
No	240	86.33
Yes	38	13.67
Diastolic hypertension		
No	236	84.89
Yes	42	15.11
Most recent (CD4+ cell count cells/ml)		
< 200	58	27.62
≥ 200	152	72.38
Viral load (copies/ml)		
< 1000	109	86.51
≥ 1000	17	13.49
ART Regimen		
TDF/3TC/EFV	271	97.48
TDF/3TC/ATV/r	135	2.16
Random blood sugar		
< 11.1 mmol/l	275	98.92
> 11.1 mmol/l	3	1.08
Protein present in urine		
≥ 2+ (present)	10	3.6
< 2+ (absent)	268	96.4
Nitrites in urine		
Positive	4	1.44
Negative	274	98.56
Hematuria present		
Present	7	2.52
Absent	271	97.48
Glucose in urine		
Present	2	0.72
Absent	276	99.28

### Electrolyte abnormalities among study participants (Table 3)

Ten (3.59%) had hypophosphatemia, 11(4.44%) had hyperphosphatemia, 15(5.26%) had hyperkalemia, 3(1.32%) had hypokalemia, 43 (15.44%) had hypocalcaemia and 2 (0.67%) had hypercalcemia.

### Prevalence of renal dysfunction among study participants

Of the 278 subjects, **7** were found to have eGFR < 60 ml/min/1.73m^2,^ ten [10] patients had proteinuria (but no glycosuria), **2** patients had glycosuria (but no proteinuria). Therefore prevalence of renal dysfunction was 2.52% (7/278). (See Table 4).

**Table 3:** Electrolyte abnormalities among study participants receiving TDF based ART ≥6 months at Mulago Communicable Disease Clinic

Parameter	Frequency(n)	Percentage (%)
Phosphate(0.97–0.45 mmol/l)		
Hypophosphatemia	10	3.70
Normal	255	91.85
Hyperphosphatemia	12	4.44
Potassium (3.5–5.0 mmol/l		
Hypokalemia	4	1.32
Normal	260	93.42
Hyperkalemia	15	5.26
Calcium (2.2–2.7 mmol/l)		
Hypocalcaemia	43	15.44
Normal	233	83.89
Hypercalcemia	2	0.67

**Table 4:** E*GFR* calculated using the CKD-EPI formula to determine renal dysfunction among study participants on TDF for ≥6 months at Mulago Communicable Disease Clinic

eGFR in ml/min/1.73m^2^	Frequency (n)	Percentage (%)	[95%Conf.Interval]
< 60	7	2.52	(1.19–5.21)
≥ 60	271	97.48	(94.72–98.80)
eGFR values for different categories			
15–29	1	0.36	
30–59	6	2.16	
60–89	51	18.35	
≥ 90	220	79.14	

## Discussion

We aimed to determine the prevalence of renal dysfunction among HIV infected patients receiving TDF attending Mulago Communicable Disease Clinic in Uganda. Defining renal dysfunction as eGFR < 60 mL/min/1.73 m^2^ or proteinuria of ≥2^+^, with glycosuria on dip stick urinalysis with a normal blood sugar. We found a low prevalence of 2.52%. This is in agreement with other studies that show that use of TDF is associated with modest renal dysfunction*.*

Udeme et al *2018* in a systematic review and meta-analysis found an overall prevalence of CKD as 6.4%. Chronic kidney disease was defined as eGFR < 60 ml/min using any of the three formulas (i.e. Cockcroft, MDRD and CKD-EPI). In this study contributing factors included WHO region, hypertension and Diabetes Mellitus. *Reid* et al *2008* in a study (DART trial) done in Uganda and Zimbabwe, noted a prevalence of 7% among patients on ART, followed up for 96 weeks. Majority (74%) of the patients were receiving TDF based therapy. In this study Glomerular filtration rate was assessed using the Cockcroft gault formula which has its limitations.

The variability of different prevalence rates in the studies is attributable to; the different definitions of renal dysfunction, different methods used to estimate GFR and patient characteristics (age, ART regimen, advanced HIV and presence/absence of other co-morbidities) [5, 10, 19].

The low prevalence in our study could be explained by the small sample size, being an outpatient clinic receiving not very critically sick patients, the possible effect of ART improving kidney function [20] and use of the CKD-Epi formula which is a more accurate method of assessing eGFR in asymptomatic patients compared to other formulas [17].

We assessed three serum electrolytes in this study namely; Potassium, Calcium and Phosphate. Albumin and vitamin D levels were not assessed. We noted mainly Hypocalcaemia (15.44%), Hyperkalemia (15%) and hypophosphatemia (3.59%).Hypocalcaemia has been found at a higher prevalence in other studies among HIV infected patients compared to the general population. Vitamin D deficiency is very common among HIV patients and may be the most reasonable explanation [21, 22]. TDF may cause vitamin D deficiency through impairment of tubular dysfunction leading to decreased 1-alpha hydroxylation of vitamin D and lower tubular reabsorption of vitamin D binding protein. There are also studies that have found an association with efavirenz use and low vitamin D levels which could also potentially explain the hypocalcaemia since 97% of their patients were on efavirenz.

For patients with hypophosphatemia, data is similar to studies elsewhere that demonstrate that TDF is associated with Proximal tubulopathy manifesting as Fanconi syndrome [20, 23]. Rarely all the components of FS occur in one patient. Because of the wasting of Phosphate, potassium and amino acids in urine they tend to be low in serum. Hypophosphatemia could have a dual origin: decreased proximal reabsorption of phosphate and decreased vitamin D activation. Another possible cause of low phosphate in serum is the possibility of Vitamin D deficiency that is prevalent in HIV infected patients [22, 23]. Hyperkalemia may be explained by the possibility of these patients having had acute kidney injury, chronic kidney disease and medications e.g.Cotrimoxazole [5, 24]. In this study majority of the patients were asymptomatic, though not enough information as regards symptoms was collected.

Proteinuria is an important marker of renal dysfunction, however for this study we tried to identify an operational definition that could in cooperate TDF nephrotoxicity. Other studies that have looked at proteinuria have given higher values of renal dysfunction. And this also helps capture the decline in renal dysfunction early to mitigate progress to ESRD.

Our study had some limitations; the small sample size (as a result we were unable to assess factors associated with renal dysfunction), missing information for some patients (e.g. CD4 and Viral load) which were not done/documented by primary health care giver and no baseline renal function to assess change over time (routine testing of renal function tests was no longer being done by the primary health care giver (findings from the DART trial) and we had limited time for the study to check the baseline levels). The results we got will form as baseline for future reference.

## Conclusions

The prevalence of renal dysfunction among HIV infected patients receiving TDF was low at 2.52% and hypocalcaemia was the most common electrolyte abnormality noted. Renal dysfunction occurs at a low rate in our settings however when assessed should include serum electrolytes.

## Supplementary information

**Additional file 1.** The datasets generated and/or analyzed during the study are available in excel format and uploaded as supplemental information.

## Data Availability

All data generated or analysed during this study are included in this published article [and its supplementary information files].

## Abbreviations

AKI: Acute kidney injury
ART: Antiretroviral therapy
CKD: Chronic kidney disease
CKD: EPI Chronic Kidney Disease Epidemiology Collaboration
EGFR: Estimated glomerular filtration rate
FS: Fanconi syndrome
PLHIV: People living with HIV
TDF: Tenofovir disoproxil fumarate
